# Suicide attempts and emergency room psychiatric consultation

**DOI:** 10.1186/s12888-015-0392-2

**Published:** 2015-02-05

**Authors:** Patrizia Zeppegno, Carla Gramaglia, Luigi Mario Castello, Fabrizio Bert, Maria Rosaria Gualano, Francesca Ressico, Isabella Coppola, Gian Carlo Avanzi, Roberta Siliquini, Eugenio Torre

**Affiliations:** Institute of Psychiatry, Università degli Studi del Piemonte Orientale, via Gnifetti, 8, 28100 Novara, Italy; Emergency Medicine, Department of Translational Medicine, Università degli Studi del Piemonte Orientale, Via Solaroli 17, 28100 Novara, Italy; Department of Public Health and Paediatric Sciences, Università degli Studi di Torino, via Santena 5/bis, 10126 Torino, Italy

**Keywords:** Emergency room, Suicide attempt, Psychiatric symptoms, Referral

## Abstract

**Background:**

Suicidal behaviours are major public health concerns worldwide. They are associated with risk factors that vary with age and gender, occur in combination, and may change over time. The aim of our study was to investigate how frequently patients visiting a hospital emergency room (ER) require a psychiatric consultation for attempted suicide, and to outline the characteristics of this population.

**Methods:**

Determinants of emergency room visits for psychiatric reasons were studied prospectively from 2008 to 2011 at the “Maggiore” Hospital in Novara.

**Results:**

280 out of 1888 patients requiring psychiatric consultation were referred to the ER because of suicide attempt. Suicide attempters were more often female. The rate of suicide attempters among Italian people was 14.2%, compared to 19.5% in foreigners. Subjects living with parents or own family and those having a permanent job had a higher frequency of suicide attempt. Suicide attempts were more frequent among patients with a history of psychiatric disorders; nonetheless, suicide attempts were more common among those who had not previously been hospitalized in a psychiatric ward or were not under the care of a psychiatrist. The multivariate analysis found that female gender was a risk factor for suicide attempt, while being in the colder months of the year and, surprisingly, unemployment were protective factors.

**Conclusions:**

A better understanding of patients referring to the ER due to attempted suicide may allow the identification of at-risk subjects and the implementation of targeted treatment approaches.

## Background

Suicide is a significant public health problem. Suicide attempts, defined as self-inflicted, potentially injurious behaviours with a nonfatal outcome, and with evidence (either explicit or implicit) of intent to die [1], are extremely prevalent. About 1 million individuals die by suicide every year, and the mortality rate for suicide is 14.5/100,000 people [2,3]. It is estimated that for each adult who died of suicide there are likely to be more than 20 others who made one or more suicide attempts [4].

Attempted suicide is usually the last step of a sequence of processes starting from death wishes, suicidal ideation, suicidal contemplation, suicidal behaviours [5,6]. Therefore, suicidal ideation, defined as thoughts of self-harming or -killing [7], is a significant marker for suicide attempts [8]. Suicidal ideation and behaviour are associated with many risk factors that can be grouped into areas that span across societal (e.g. discrimination), relationship (e.g. sense of isolation and lack of social support) and individual risk factors (e.g. job or financial loss, biological and genetic factors, illness), which vary with age and gender, occur in combination, and may change over time [9]. For example, older adults are at greater risk for suicide than any other age group [10]. Suicide is more common among males, while attempted suicide is more frequent among females [11-13]; there are many theories about these gender differences, spanning from the choice of suicide methods [14] to the relationship with hormonal factors [15,16].

Depression, other mental disorders, history of suicidal behaviour and substance use disorders are important risk factors for suicide, with one or more of these being present in over 90% of all completed suicides [17]. In particular, a history of suicidal behaviour is an extremely strong risk factor for completed suicide. Those who attempt suicide are 38 times more likely than others to eventually go on to complete suicide, and previous studies found that attempted suicides are three to ten times more numerous than completed suicides [18-21]. Suicide risk should be studied also in relation to level of psychiatric treatment. A recent research [22], which anyway was focused on suicide rather than suicide attempt, has found that among psychiatric treatments in the previous year, including “no treatment”, “medicated”, “outpatient contact”, “psychiatric emergency room contact”, or “admitted to psychiatric hospital”, the last two were those with the strongest association with suicide (OR 27.9 and OR 44.3, respectively).

Although Italy does not fill in a record of suicide attempts at the time of hospitalization, about 70% of self-harms referred to the Emergency Room (ER) are the result of a suicide attempt [23]. We focused our research on suicide attempters, who may be at greater risk of repeating the attempt [18-21] and to eventually go on to complete suicide; and since suicidal patients often meet mental health care for the first time in the ER, we assessed the population of suicide attempters referring to the ER. The current research was conducted in Piedmont, which is an Italian region at high risk for suicide [21,24,25], particularly as regards its North-Eastern area, where the Azienda Ospedaliero Universitaria (AOU) Maggiore della Carità Hospital is located. This is the second largest general hospital in Piedmont, with a large user base.

The aim of the present study was to investigate the rates of suicide attempts in psychiatric ER users (i.e. those patients referring to the hospital ER, for whom a psychiatric consultation is required) at the AOU Maggiore della Carità Hospital in Novara. It was also intended to describe the differences between patients with and without a suicide attempt referring to the psychiatric ER with regard to demographic features, type of referral to the ER, clinical and social issues, nationality, temporal factors, method of the attempt, and clinical outcome.

## Methods

The Emergency Department (ED) of the Maggiore della Carità Hospital, Novara, includes a high specialization level Emergency Room (ER) receiving about 60,000 adult people per year. The Maggiore della Carità Hospital is the second largest general hospital in Piedmont, the main referral hospital for all North-Eastern Piedmont, and its catchment area is representative of the whole region. In our hospital all acute patients who are assessed at the ER are evaluated by the emergency medicine physician according to a priority code attributed by the nurse through a triage evaluation. The emergency physician requires the consultation of psychiatrists, as well as of other specialists, after a preliminary assessment of the patients, considering patients’ clinical features, and according to the hospital guidelines for the ER. For instance, according to these guidelines, every suicide attempt is referred to the psychiatrist. Anyway, of course, those patients who have committed a suicide attempt (for instance, shooting, defenestration, jumping from high places) requiring other first-line, life-saving treatments, are not referred to a psychiatrist in the ER setting, but rather at a later time, when their medical condition is stable. For this reason, these patients were not included in the current study.

We recruited consecutive patients referring to the ER of the Maggiore della Carità Hospital during the period 2008–2011. To be involved in the study, the only inclusion criterion was being referred to psychiatric assessment after ER triage. Hence, from all ER referrals, we obtained a sample of 1888 ER patients who underwent psychiatric evaluation. The psychiatric assessment of patients was performed by experienced psychiatrists with a clinical interview, including the assessment of suicidal intent, suicidal behaviours and attempts. For each patient, a data sheet was filled in by the psychiatrist, in order to gather demographic features, type of referral to the ER, psychiatric history and present clinical issues, type of acute intervention delivered to patient, and psychiatric admission rates.

The patients’ names were only registered in the data sheet in order to assess possible multiple referrals to the ER. Patients were then given a numerical code which was registered in a separate datasheet whose access is restricted to the researchers involved.

No exclusion criteria were applied: all patients referring to the ER and requiring a psychiatric evaluation were taken into consideration. Nevertheless, we should bear in mind that patients aged <16 years refer to a separate paediatric ER.

For the analyses, the psychiatric ER referrals (n = 1888) were later subdivided into two groups according to suicide attempts: those referred to the ER for a suicide attempt (n = 280), and those referred for different psychiatric reasons (n = 1608).

The project was approved by the Institutional Review Board of the Università del Piemonte Orientale as part of the research duties of the Psychiatry Institute. Informed consent was obtained from patients.

### Statistical analysis

Descriptive statistics were performed using frequencies, percentages, frequency tables for qualitative variables, mean with Standard Deviation (SD), and Min-Max values for quantitative variables. In order to evaluate the differences in proportions between groups (suicide attempt yes/no) the chi-squared test was used, while differences between groups for continuous variables were assessed through a t-test. Then, a multivariate analysis was performed using logistic regression in order to assess the potential predictors of attempted suicide. The covariates included in the final model were selected through the Hosmer and Lemeshow procedure, by inserting variables with a univariate p-value <0.25 as the main criterion [26]. Results are expressed as Odds Ratio (OR) with 95% Confidence Intervals (95% CI). Statistical significance level was set at p ≤ 0.05. Statistical analyses were performed with STATA 11 (Stata Corp., 2011).

## Results

### Descriptive data

During the study period (from 2008 to 2011) 230,257 patients were evaluated in the ER; 1888 of these patients underwent psychiatric evaluation in the ER. Our sample thus constituted 823 men (43.6%) and 1065 women (56.4%), with a mean age of 44.9 (44.2 - 45.7) years; 1652 patients were Italian (87.5%) and 236 were foreign (12.5%).

As far as the accommodation status is concerned, 455 patients lived alone (26.2%), 1163 with immediate or own family (67.0%), and 119 lived in therapeutic communities/social services (6.8%). With respect to marital status, 627 subjects were married (36.3%), while 1100 were not (63.7%).

Of the sample 74.2% (n = 1243) had a primary or middle school education, while only 25.8% (n = 432) had a high school qualification or a degree.

The majority of patients did not work: 73.4% were unemployed and 0.7% were retired or disabled.

n = 1187 patients (63.1%) had a history of psychiatric disorders and 966 (51.3%) were under psychopharmacological treatment.

Table 1 summarizes the main psychiatric symptoms at the psychiatric consultation in the ER, both for suicide attempters and non-attempters.

**Table 1:** **Symptoms according to DSM IV-TR in the sample of ER patients referred for psychiatric consultation**

	**Suicide attempters**		**Non attempters**	
**Symptoms**	**n**	**%**	**N**	**%**
Anxiety Disorders	62	22.1	596	37.1
Absence of psychiatric symptoms	79	28.2	225	14.0
Substance Use Disorders	34	12.1	225	14.0
Mood Disorders and Bipolar Disorders	65	23.2	157	9.7
Schizophrenia and other Psychotic Disorders	6	2.2	137	8.5
Neurocognitive Disorders	18	6.4	112	7.0
Psychomotor agitation, excluding intoxication, withdrawal syndrome or dementia	10	3.6	101	6.3
Other (e.g. EPS, neurologic symptoms)	6	2.2	54	3.4
Total	280	100	1608	100

#### Comparison between suicide attempters and non-attempters

Out of the 1888 patients requiring psychiatric consultation 280 (14.8%) were referred to the ER because of a suicide attempt. Their mean age was 42.67 ± 16.37 years old.

The comparison between suicide attempters and non-attempters revealed some significant differences both in socio-demographic variables and in clinical ones. See Table 2 for details.

**Table 2:** **Description of the sample according to suicide attempt (n = 1888)**

		**Suicide attempt**		
		**Yes** ***% (n)***	**No** ***% (n)***	**p**
**Gender**	Male	C 34.6 *(97)*	C 45.2 *(726)*	**<0.001**
		R 11.8	R 88.2	
	Female	C 65.4 *(183)*	C 54.8 *(881)*	
		R 17.2	R 82.8	
**Nationality**	Italian	C 83.6 *(234)*	C 88.2 *(1417)*	**0.032**
		R 14.2	R 85.8	
	Foreign	C 16.4 *(46)*	C 11.8 *(190)*	
		R 19.5	R 80.5	
**Living Arrangement**	Alone	C 21.8 *(58)*	C 27.0 *(397)*	**0.009**
		R 12.7	R 87.3	
	With parents or own family	C 74.4 *(198)*	C 65.6 *(965)*	
		R 17.0	R 83.0	
	Community/social services	C 3.8 *(10)*	C 7.4 *(109)*	
		R 8.4	R 91.6	
**Marital status**	Not Married	C 55.3 *(147)*	C 65.2 *(953)*	**0.002**
		R 13.4	R 86.6	
	Married	C 44.7 *(119)*	C 34.8 *(508)*	
		R 19.0	R 81.0	
**Educational level**	Primary or Middle School	C 72.5 (185)	C 74.5 (1058)	0.5
		R 14.9	R 85.1	
	High School or Degree	C 27.5 (70)	C 25.5 (362)	
		R 16.2	R 83.8	
**Occupation**	Employed	C 34.9 (89)	C 24.4 (354)	**<0.001**
		R 20.1	R 79.9	
	Unemployed	C 65.1 (166)	C 74.8 (1087)	
		R 13.2	R 86.7	
	Retired/Invalid	C 0.0 (0)	C 0.8 (12)	
		R 0.0	R 100.0	
**History of psychiatric disorders**	Yes	C 55.9 (156)	C 64.3 (1031)	**0.007**
		R 13.1	R 86.9	
**Under the care of a psychiatrist**	Yes	C 43.4 (121)	C 52.7 (845)	**0.004**
		R 12.5	R 87.5	
**Treated with psychiatric drugs**	Yes	C 51.3 (143)	C 58.3 (934)	**0.028**
		R 13.3	R 86.7	

C = column percentage.

R = row percentage.

Patients with suicide attempt were more often female (65.4%, n = 183) than male (34.6%, n = 97) (p < 0.001). Of the suicide attempters 83.6% (n = 234) were Italian, while 16.4% were foreign (n = 46). The rate of suicide attempters was 14.2% among the Italian people requiring a psychiatric consultation in the ER, compared to 19.5% in foreigners (p = 0.032).

As far as the living arrangement is concerned, 58 subjects lived alone (21.8%), 198 with their immediate or own family (74.4 %), 10 lived in a community or were under the care of social services (3.8%). Specifically, suicide attempt was more common in those living with parents or own family, compared to those living alone or in a community (p = 0.009). Among suicide attempters, 147 patients were not married (55.3%), and 119 were married (44.7%). The rate of married people who had attempted suicide was 19% vs 13.4% of those not married (p = 0.002).

No significant difference was found as far as the educational level is concerned between the two groups of attempters and non-attempters. As regards the occupational status, although in absolute figures most patients were unemployed (65.1%, n = 166), there was a higher frequency of suicide attempts among those who had a permanent job (20.1% vs 13.2%; p < 0.001).

With respect to the clinical history, suicide attempts were more frequent among patients who had a history of psychiatric disorders (55.9%, n = 156 vs no history 44.1%, n = 123; p = 0.007). Yet, suicide attempts were more frequent among those who had never been hospitalized in a psychiatry ward in the past (69.1%, n = 192 vs previously hospitalized 30.9%, n = 86; p = 0.01) and those who were not under the care of a psychiatrist (56.6%, n = 158 vs under psychiatric treatment 43.4%, n = 121; p = 0.004).

#### Type of suicide attempt

As far as the type of attempt is concerned, we observed a greater frequency of drug poisoning (69.3%) vs cutting by sharp objects (17.1%) or other methods (13.6%). Interestingly, men are more likely to cut themselves than women (27.8% vs 11.5%), while women seemed to have more propensity to self-poisoning (76.5% vs 49.5%) with a p-value lower than 0.001. As far as the type of drug used is concerned, benzodiazepines or barbiturates (47.9%), polydrugs (22.4%) and non-psychiatric drugs (15.10%) were the most taken drugs.

#### Outcome of ER referral

As regards the type of intervention offered in the ER by the consulting psychiatrist, n = 234 patients (83.6%) only required a psychiatric interview, while 30 subjects (10.7%) were also administered an acute therapy; 10 patients (3.6%) were prescribed a psychopharmacological treatment start-up and 2.1% (n = 6) of the sample underwent an adjustment of the ongoing treatment. As regards the treatment prescribed, 67.7% received or were prescribed benzodiazepines, 14.7% typical antipsychotics, 5.9% benzodiazepines plus antipsychotics, and 11.8% other drugs.

As far as the outcome of the psychiatric ER referral is concerned, n = 97 patients (34.6%) who attempted suicide were admitted to the Psychiatry Ward, and 16 patients (5.7%) to the Psychiatry Day Hospital or other outpatient services. 118 patients (42.1%) were directly dismissed from the ER after the psychiatric consultation while 35 patients (12.5%) were dismissed after a short period of clinical observation. 9 patients (3.2%) were admitted to other Wards than the Psychiatry Ward or needed a consultation by another specialist. Eventually, 5 patients (1.8%) self-discharged from the ER.

#### Time of referral, seasonality and trend through the years

In the population we assessed (ER patients referred to psychiatric consultation), we observed a total suicide attempts rate of 0.025, with a slight, but not significant, increase through the years considered. Analyzing suicide attempts by year and season (Table 3), we observed no significant difference among the years of the period investigated, yet we found a greater frequency of episodes during the warmer months of the year (from April to September), with a statistically significant difference (56.1% vs 43.9%; p = 0.02).

**Table 3:** **Suicide attempt according to seasonality and year**

		**Suicide attempt**		
		**Yes**	**No**	**p**
**Seasonality**	Warm months (April – September)	63.6	56.1	**0.02**
	Cold months (October – March)	36.4	43.9	
**Year**	2008	26.4	29.1	0.16
	2009	22.9	27.0	
	2010	28.2	25.9	
	2011	22.5	18.0	

We then performed a cross-tabulation between attempted suicide and time of referral to the ER (count within time of referral: 14.9% vs 14.6% of suicide attempts in day and night, respectively; count within suicide attempt: 69.3% vs 30.7% in day and night, respectively), but failed to find any significant result (χ2 test, p = 0.845).

#### Multivariate analysis

Female gender was found to be a risk factor for suicide attempt, with an OR of 1.51 (p = 0.005). We also found that being in the colder months of the year (from October to March) was a protective factor for attempted suicide (OR = 0.75; p = 0.043), as well as being unemployed (OR = 0.68; p = 0.013) (see Table 4).

**Table 4:** **Potential predictors of suicide attempt in patients admitted to emergency department**

	**Adjusted OR**	**95% CI**	**p**
**Gender: Female**	1.51	(1.13 - 2.02)	**0.005**
**Nationality: Foreigner**	1.30	(0.87 - 1.94)	0.2
**Age**	0.99	(0.98 - 1.00)	0.2
0-18	1		
19-29	1.07	(0.48 – 2.40)	0.9
30-39	0.85	(0.39 – 1.84)	0.7
40-49	1.03	(0.48 – 2.22)	0.9
50-59	0.99	(0.45 – 2.22)	0.9
60-69	1.06	(0.45 – 2.49)	0.9
70+	1.21	(0.53 – 2.74)	0.7
**Seasonality: Cold months**	0.75	(0.57 - 0.99)	**0.043**
**Accomodation status**			
Alone	1 (Ref)	-	-
Own family or immediate family	1.09	(0.75 - 1.58)	0.7
Therapeutic community, retirement home, prison	0.69	(0.34 - 1.42)	0.3
**Marital status: Not Married**	1.35	(0.98 - 1.87)	0.07
**Occupation**			
Employed	1 (Ref)	-	-
Unemployed	0.68	(0.50 - 0.92)	**0.013**
Retired/Disabled	--		
**History of psychiatric disorders**	0.80	(0.52 - 1.23)	0.3
**Under the care of a psychiatrist**	0.85	(0.56 - 1.30)	0.5

Ref = reference category.

## Discussion

280 out of 1888 patients requiring psychiatric consultation were referred to the ER because of a suicide attempt. In our sample, we found statistically significant differences between suicide attempters and non-attempters in several variables, including gender, employment, migrant condition, living accommodation, marital status, and history of psychiatric disorders. Moreover, female gender and having a permanent job were found to be risk factors for suicide attempts.

Attempted suicide is influenced by multiple factors and epidemiologic and clinical information are key issues for the development of a schedule for treatment and prevention. The first contact with suicide attempters usually occurs at the ER, which therefore represents the best place to obtain these data.

As far as gender is concerned, we found that attempted suicide is more frequent among females, with a ratio of almost 2:1, in accordance with the literature. In developed countries, the completed suicides are two to four times more frequent among men [11,12] while suicide attempts are two to three times more frequent among women [13,27]. The most frequent methods for attempting suicide in our sample were drugs poisoning (69.3%) and cutting by sharp objects (17.1%).

Although literature reports evidences of a high risk of suicide in unemployed subjects [28,29], in our study we observed that attempted suicide is more frequent among those who have a job. Our findings might be associated with the current Italian economic scenario, and to emerging difficulties, especially in the middle-class, in keeping an income to adequately maintain their family’s standard of living [30,31]. Moreover, when comparing our results with the current literature, we should consider that some studies are focused on suicide rather than on suicide attempts and suicidal behaviours, and that our study labels as “employed” those who are under redundancy fund or who are employed de facto, but do not actually work. Regarding redundancy fund, it should be noted that in Italy workers have the right to the provision of financial support sufficient to meet their needs in case of involuntary unemployment (for instance because of difficulties or even bankrupt of their factories or companies); this support is supplied for a maximum of 3 years, but after that the individual is no longer supported.

We also observed a greater frequency of attempted suicides among those who live with parents or own family, and among married individuals. To interpret this result which seems to suggest that individuals living in apparently more favourable conditions may be at greater risk of attempting suicide, we should consider again that the middle-class is hindered by the current socio-economic context. Moreover, while most literature describes loneliness as a possible risk factor for suicide, it is possible that relational problems have some correlation with suicide attempt, as well. Wu et al. [32], for instance, found that female suicide attempters admitted to psychiatric ER assessment in a general hospital reported more family relationship problems than males, who more commonly reported economic problems.

In our study we found a correlation between suicide attempt and being a migrant. Some authors agree that immigrants suffer from more neuroses and attempt suicide more often than natives [33-35]. Such differences might be explained by the stress caused by the migration process, including perceived lack of support [34], precarious socio-economic conditions [36], ethnic discrimination by the host population [37], and acculturation [38].

As far as a seasonal variation in attempted suicide is concerned, we observed a significantly greater frequency of episodes during the warmer months (spring and summer months) of the year and a greater frequency, albeit non-significant, of episodes during the daytime, in accordance with the literature, postulating a positive association between sunshine, temperature and self-injury behaviours [39-43].

As regards patients’ clinical history, we observed that suicide attempts were more frequent among those who had a history of psychiatric disorders, but were not under the care of a psychiatrist. The lack of compliance with appropriate therapies, adequate follow-up and rehabilitation for psychiatric disorders, may increase the risk of suicide attempts in psychiatric patients who are already at risk, for instance those with schizophrenia and mood disorders.

Finally, we found that female gender and a permanent job were risk factors for suicide attempts, as well as being in the warmer months of the year. Despite being in contrast with most data in the literature, the finding that suicide attempters were better integrated both in private and occupational life, compared to patients referring to a Psychiatric ER for other emergencies, has already been reported [44].

Regarding the outcome of ER referral, 34.6% of patients were admitted to the psychiatric ward, while 42.1% were dismissed from the ER. These data are in agreement with the literature [45,46].

Some limitations of this study should be underscored. First, data should be carefully interpreted by taking into account that the sample enrolled in the present research could be considered as representative only of the population living in Piedmont [47]. It could be very interesting to perform future multicentre studies in order to describe the Italian situation about this topic, considering that North, Centre, and South Italy are fairly different as far as socio-economic variables are concerned. Second, as our study involved psychiatric ER referral, it may underestimate the suicide attempts rate, because patients committing severe suicide attempts which require an intensive care treatment do not undergo a psychiatric evaluation at the ER but might be hospitalized at our ward after stabilization of their clinical picture. Hence, such patients were not included in our sample and this may cause an underestimation of the overall number of suicide attempters in our catchment area. Moreover, since this study was performed in a hospital setting, those suicide attempters who did not refer to the hospital were not included.

Last, although similar studies on emergency room registers have been performed in other hospitals from other countries [44,46,48,49], they share flaws similar to our own and of course a limit of this field of research is the use of different approaches to assessment which may limit the generalizability of results. Nonetheless, all of these studies offer useful information about the possible correlates of suicidal behaviour and to the approach to at-risk patients in an ER setting. Since clinical features and ER policies and treatment strategies are a major determinant of suicidal patients’ referral to a psychiatric ward, all research increasing knowledge about possible correlates of suicide attempts should be welcome.

## Conclusions

Suicide is an important public health problem. It is estimated that an ER consultation related to self-injury is the highest risk factor for a future completed suicide [50]. Recently, the need of a better identification of the needs and features of suicide attempters has been underscored, considering the high rate of psychiatric morbidity and interpersonal problems of these patients, their poor adherence to psychiatric outpatient aftercare and ER clinicians’ perception of gaps in referral skills [51,52].

In our study we observed that being a female, living in apparently more favourable conditions, or being a migrant are associated with suicide attempts, as well as being in the warmer months of the year, or being affected by a non-adequately treated psychiatric disorder. Moreover, according to our results, the female gender, a permanent job, and being in the warmer months of the year are risk factors for suicide attempts. A better understanding of ER patients referred due to attempted suicide may allow the identification of at-risk features and may encourage the development of prevention strategies and models of intervention which can support primary care physicians, ER clinicians as well as psychiatrists. For instance, suicide attempters showing the features and risk factors described above might be supported by a targeted intervention during hospitalization and after discharge, accounting for the problematic areas identified in each case. The approaches to interpersonal/relational problems, psychiatric morbidity and psychosocial issues are different and may be complementary (psychotherapy, medication, psychosocial support) and should be tailored to each individual.

Last, studies involving a population representative of the whole Nation are warranted to increase the generalizability of our findings.
